# Implications of the licensure of a partially efficacious malaria vaccine on evaluating second-generation vaccines

**DOI:** 10.1186/1741-7015-11-232

**Published:** 2013-10-30

**Authors:** Freya JI Fowkes, Julie A Simpson, James G Beeson

**Affiliations:** 1 Macfarlane Burnet Institute of Medical Research, 85 Commercial Road, Melbourne, Victoria 3004, Australia; 2 Centre for Molecular, Environmental, Genetic and Analytic Epidemiology, University of Melbourne, 207 Bouverie Street, Melbourne, Victoria 3010, Australia; 3 Department of Epidemiology and Preventive Medicine and Department of Infectious Diseases, Monash University, Commercial Road, Melbourne, Victoria 3004, Australia; 4 Department of Microbiology, Monash University, Melbourne, Victoria, Australia; 5 Department of Medicine, University of Melbourne, Melbourne, Victoria, Australia

**Keywords:** Malaria, Vaccine, Clinical trials

## Abstract

**Background:**

Malaria is a leading cause of morbidity and mortality, with approximately 225 million clinical episodes and >1.2 million deaths annually attributed to malaria. Development of a highly efficacious malaria vaccine will offer unparalleled possibilities for disease prevention and remains a key priority for long-term malaria control and elimination.

**Discussion:**

The Malaria Vaccine Technology Roadmap’s goal is to 'develop and license a first-generation malaria vaccine that has protective efficacy of more than 50%’. To date, malaria vaccine candidates have only been shown to be partially efficacious (approximately 30% to 60%). However, licensure of a partially effective vaccine will create a number of challenges for the development and progression of new, potentially more efficacious, malaria vaccines in the future. In this opinion piece we discuss the methodological, logistical and ethical issues that may impact on the feasibility and implementation of superiority, non-inferiority and equivalence trials to assess second generation malaria vaccines in the advent of the licensure of a partially efficacious malaria vaccine.

**Conclusions:**

Selecting which new malaria vaccines go forward, and defining appropriate methodology for assessment in logistically challenging clinical trials, is crucial. It is imperative that the scientific community considers all the issues and starts planning how second-generation malaria vaccines will advance in the advent of licensure of a partially effective vaccine.

## Background

Malaria caused by *Plasmodium* spp. is a leading cause of morbidity and mortality, with approximately 225 million clinical episodes and >1.2 million deaths annually attributed to this parasitic disease [1,2]. The Malaria Vaccine Technology Roadmap states that the first landmark goal, to be achieved by 2015, is to 'develop and license a first-generation malaria vaccine that has protective efficacy of more than 50% against severe disease and death and last longer than 1 year’ [3]. To date, malaria vaccine candidates have only been shown to be partially efficacious. The most advanced candidate, RTS,S, is a *Plasmodium falciparum* pre-erythrocytic vaccine currently undergoing phase III trials that has only shown moderate to modest efficacy. The preliminary vaccine efficacy (VE) estimates of RTS,S for the first 6,000 infants and young children (aged 5 to 17 months) for 12 months of follow-up were 56% (95% CI 51 to 60) for clinical malaria and 47% (95% CI 22 to 64) for severe malaria due to *P. falciparum*[4]. However, RTS,S VE was recently reported to be lower in younger infants (aged 6 to 12 weeks); 31% (95% CI 24 to 38) for clinical malaria and 37% (95% CI 5 to 58) for severe malaria for the same length of follow-up [5]. Some other vaccines, targeted at the *P. falciparum* blood stage, have shown limited to no protective efficacy in placebo-controlled trials [6,7], but have demonstrated partial vaccine efficacy that is allele specific (that is, the vaccine protects against infection or disease due to vaccine-like alleles, but not other alleles) when a smaller number of allele-specific endpoints were analyzed. In a trial of 400 children (aged 1 to 6 years), the blood-stage vaccine FMP2.1/AS02(A), a recombinant protein based on *P. falciparum* apical membrane antigen 1 (AMA1; 3D7 allele), showed no significant VE when all clinical episodes were considered (17%, 95%CI -9 to 37), but demonstrated an allele-specific VE of 64% (95% CI 14 to 92) against clinical malaria [6]. Another blood-stage vaccine, Combination B, comprising recombinant *P. falciparum* ring-infected erythrocyte surface antigen and 2 merozoite surface proteins (MSP1 and MSP2), demonstrated a VE of 62% (95% CI 13 to 84) against parasitemia, but no significant VE against all symptomatic malaria episodes in a trial of 120 children aged 5 to 9 years [7]. Similarly, in this trial there was evidence of allele-specific immunity targeting MSP2, with vaccinees having some protection against infections with vaccine-like MSP2 alleles, but not against non-vaccine alleles [7]. Ongoing development of AMA1 and MSP2 is now focused on overcoming the allele-specific nature of protection to provide broad coverage against different alleles [8,9].

Given the results from malaria vaccine trials to date, it is likely that a first-generation malaria vaccine will be partially efficacious and restricted to protection against *P. falciparum* malaria only, so second-generation vaccines will be needed in the future. However, licensure of a partially efficacious vaccine creates a number of challenges in the development and testing of second-generation vaccines. In this opinion piece, we discuss the methodological issues and feasibility of second-generation vaccine trials in the advent of the licensure of a partially efficacious malaria vaccine.

## Discussion

### Testing a second-generation malaria vaccine in clinical trials

A second-generation malaria vaccine may seek to improve on a first-generation vaccine by being more efficacious, having longer duration, including additional *Plasmodium* spp. or having more favorable logistics compared to the previous generation vaccine. New malaria vaccines may target single, or multiple stages of the *P. falciparum* lifecycle (pre-erythrocytic, blood and/or sexual stage), or another species such as *P. vivax*, and each target may have multiple allelic variants to consider for inclusion in a new malaria vaccine. These potential targets may be included in standalone vaccines (for example, a more efficacious vaccine targeting the same lifecycle stage, those targeting other lifecycle stages or other *Plasmodium* spp.) or be added to the first-generation vaccine in combination to address the various gaps in the lifecycle, for example, pre-erythrocytic + gametocyte antigen (to enhance transmission blocking activity), pre-erythrocytic + blood-stage antigen (to enhance protection against clinical illness, for example, merozoite antigens). Alternatively, the VE of the new malaria vaccine may be similar to the VE of the first-generation vaccine but it may be more attractive because it is cheaper to make, easier to administer (for example, skin patch, oral), more stable (for example, does not require cold storage) or have a more favorable immunization regimen.

Licensure of the first malaria vaccine creates ethical challenges for performing placebo-controlled trials for the evaluation of second-generation vaccines; placebo-controlled trials are typically only acceptable if no standard treatment exists. A placebo-controlled trial may be justified in specific circumstances, such as where a second-generation malaria vaccine has a different objective (for example, elimination vs morbidity reduction) or will be administered to a different target population (for example, adults, pregnant women). However, performing placebo-controlled trials is likely to be considered unethical in groups included within the first-generation vaccine licensed indications, particularly if the malaria vaccine is adopted as a national policy. It is most likely, then, that second-generation vaccines will have to be assessed relative to a first-generation malaria vaccine (as an active control) in the context of superiority, non-inferiority and equivalence trials, whereby the VE of a new malaria vaccine will be determined to be better than or just as good as the first-generation vaccine. The feasibility of such trials is currently unclear and there are concerns in the malaria community that the sample sizes required for these trials would be very large and potentially unfeasible or difficult to fund [10]. This has certainly been the case for other vaccines in the past, such as those against *Streptococcus pneumoniae* or *Neisseria meningitides*, where the barriers of sample size have been so great that second-generation vaccines have been licensed on the basis of an accepted immunologic outcome [10]. However, comparing new malaria vaccines to current vaccine candidates using analysis of immune responses only is currently not possible because we do not know the definitive mechanisms by which current vaccine candidates protect against malaria [11], and there is no widely accepted immune correlate of protection. For the most advanced candidate, RTS,S, while there is evidence that high titers of circumsporozoite antibodies are predictive of protection [12], it is not clear whether antibody titers can be reliably used as a strong correlate of protection in vaccine development and evaluation. Additionally, we do not know the functional mechanisms (complement, antibody-dependent cell cytotoxicity, neutralization or phagocytosis) by which antibodies confer protection [13]. T cells are also thought to be an important mechanism [14], but correlations between T cells and protection against clinical outcomes in RTS,S trials have been inconsistent in published studies [13]. Furthermore, the immunological mechanisms of a new malaria vaccine (especially one targeting other lifecycle stages and species) are likely to be different to a first-generation vaccine. Alarmingly, there are currently no established validated immunological correlates of protection against malaria for any antigen [11].

Defining immunological correlates of protection against malaria is particularly imperative in the context of potential combination vaccines. Such vaccines, that combine antigens from two or more different vaccines, are often submitted for licensure on the basis of non-inferiority trials that demonstrate safety and immunogenicity profiles similar to component vaccines administered separately [15]. Combination vaccines are often proposed as a future solution that may help overcome the many ethical and regulatory issues in developing second-generation vaccines: a new antigen could be included in combination with the antigen(s) in the licensed vaccine (for example, RTS,S plus antigens). However, not knowing the immunological correlates of protection from malaria means that the assessment of second-generation malaria vaccines cannot rely on analysis of immune responses induced by the vaccine, but will most likely have to progress to superiority, non-inferiority or equivalence field trials where they will be compared to first-generation vaccine. Given the complexities, logistics and costs of performing field trials in malaria endemic areas, it is essential to identify immunological correlates and assays to enable the malaria community to carefully select and prioritize new malaria vaccines for testing in future large clinical field trials. Additional avenues should also be explored to help evaluate new vaccines prior to embarking on large and costly clinical trials. For example, testing candidate vaccines in the human challenge model (in which volunteers are experimentally infected with *Plasmodium*) may be valuable in prioritizing or refining vaccines prior to conducting large field trials.

### Superiority, non-inferiority and equivalence field trials of second-generation malaria vaccines

**Figure 1:**
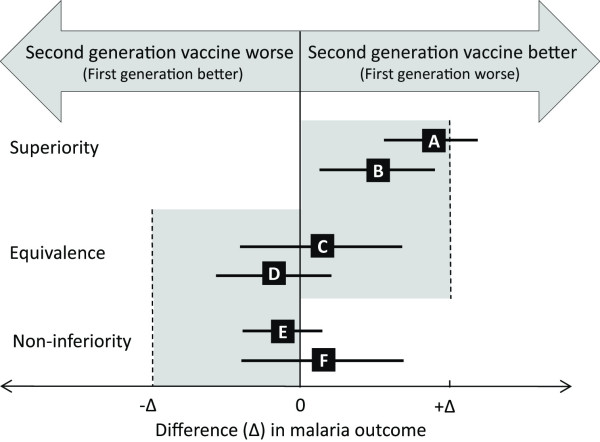
**Defining superiority, equivalence and non-inferiority in clinical trials of second-generation malaria vaccines.** Summary of the possible trial types, outcomes and considerations when testing second-generation malaria vaccines compared to a partially efficacious first-generation licensed vaccine. Error bars correspond to possible trial outcomes and indicate two-sided 95% confidence intervals (CI). Δ (the superiority margin (+Δ), non-inferiority margin (-Δ) and equivalence margin (-Δ to +Δ) can be defined by an absolute or a relative difference in actual malaria outcomes. Interpretation of trials depends on where the CI for the true difference in outcome falls relative to Δ and the null effect (0). For superiority trials, to conclude superiority, the trial effect may be bigger or smaller than Δ but the 95% CI must be above 0 (scenarios A and B). For equivalence trials, equivalence requires the CI to lie wholly within a bidirectional symmetrical equivalence margin (-Δ and Δ, scenarios C and D). If the effect estimates lie outside the bidirectional symmetrical equivalence margins, the second-generation malaria vaccine is either better or worse than the first-generation vaccine [16]. In a non-inferiority trial, the prime interest is determining whether the new malaria vaccine is no worse than the non-inferiority margin (-Δ) which, if exceeded, defines the new treatment as being inferior to RTS,S. For non-inferiority trials, if the CI lies completely to the right of the prespecified margin (-Δ) a conclusion of non-inferiority of the second-generation malaria vaccine is reached (scenarios E and F). If the CI includes -Δ it is concluded that the new malaria vaccine is inferior to the first-generation malaria vaccine.

The choice of superiority, non-inferiority or equivalence trial depends on the objective of the new malaria vaccine. A superiority trial would be suitable if the desire was to replace the first-generation vaccine with a more efficacious vaccine, and the aim would be to demonstrate that the new malaria vaccine was better by a predefined clinically accepted margin (Δ) (Figure 1). This is the same principle as a placebo-controlled trial, except the placebo is replaced with an active control. Conversely, if a new malaria vaccine was predicted to have a similar VE to the first-generation vaccine but had longer duration, was cheaper, had favorable logistics or contained additional lifecycle stages or antigens, these would provide the rationale for a non-inferiority or equivalence trial. The objective of non-inferiority and equivalence trials is to determine whether the effects of the new malaria vaccine stay within or go beyond a predefined clinically acceptable margin (Δ) relative to the standard licensed vaccine (Figure 1). After clinical trials, the new malaria vaccine may be recommended if it has a VE that is similar, but not inferior, to the first-generation vaccine. The choice of non-inferiority or equivalence trial will depend on the scientific question being asked. The hypothesis being tested in a non-inferiority trial is that the second-generation vaccine is as good as, or better than, the first-generation vaccine. Non-inferiority trials are used to show that a minimum level of efficacy has been achieved by the new vaccine. This trial would therefore be favorable for vaccines that seek to advance first-generation vaccines by being more efficacious or have longer duration. In an equivalence trial, the hypothesis being tested is that the second-generation vaccine cannot be worse than or better than the first-generation vaccine. Equivalence trials would be used to demonstrate that a new vaccine is clinically equivalent to a current vaccine in its efficacy, and they are used in the registration and approval of vaccines that have been shown to be bioequivalent.

Defining an appropriate margin of superiority, non-inferiority or equivalence to a vaccine with limited efficacy (for example, 50%) [3] is complex, both in the context of trials conducted solely for licensing purposes and for trials investigating the VE of new antigens. Careful consideration is required to define how much better the new malaria vaccine needs to be or how much lower efficacy is acceptable. If the long-term strategic goal of the Malaria Vaccine Roadmap is realized (a more efficacious second-generation vaccine with >80% VE) [3] then wide margins will be selected for determining the sample size calculation of a superiority trial (that is, >30% margin assuming VE of the first-generation vaccine is 50% [3]). But by specifying margins that are too wide (and thus conducting trials with small sample sizes) we run the risk of classifying a new malaria vaccine, with potential clinically significant benefits (for example, VE 20 percentage points higher than first-generation vaccine), as non-superior to the first-generation vaccine. This may particularly be the case for new antigens that may show some degree of VE that can be improved upon. Conversely, by being more conservative and specifying margins that are too narrow, the new malaria vaccine may be statistically significantly better than the licensed malaria vaccine, but the magnitude of the effect may be of limited benefit at the clinical and public health level.

Similar issues arise in choosing margins for non-inferiority and equivalence trials; by specifying margins that are too wide we run the risk of classifying inferior malaria vaccines as non-inferior/equivalent to first-generation vaccine, whereas by specifying margins that are too narrow we run the risk of potentially rejecting a new malaria vaccine that may provide some clinical and other benefits. Clinically acceptable margins in non-inferiority and equivalence trials of antimalarial drugs have typically ranged from 5% to 10%, but the efficacy of antimalarial treatment regimens are typically >90% [17-23]. The landmark goal of the current Malaria Vaccine Technology Roadmap is a vaccine with 50% efficacy, and the VE of the most advanced candidate RTS,S is around this level [3,4]. If we assume a VE of 50%, then a 5% to 10% absolute margin in a non-inferiority/equivalence trial would mean that the new malaria vaccine would need to have a VE of at least 40% to 45% to be considered equivalent. This raises the question of whether the malaria community would be satisfied with a second-generation malaria vaccine with efficacy of 40%? Additionally, would a 40% efficacy in the incidence of symptomatic malaria, or other relevant endpoints, be considered as clinically non-inferior/equivalent? Even if the limit is set to 45% for the new vaccine, the new malaria vaccine would be considered non-inferior/equivalent to the first-generation vaccine, despite there potentially being up to 5,000 more episodes of malaria following vaccination of 100,000 individuals, with subsequent exposure to malaria, with the second-generation malaria vaccine compared to the first-generation vaccine. While a consensus may be reached on a clinically relevant margin, should the choice of margin be influenced by the cost of the second-generation vaccine and would a wider margin of non-inferiority/equivalence be more acceptable if the second-generation vaccine is considerably cheaper and easier to deploy? The level of compromise between cost and clinical/logistical relevance in choice of margin is yet to be determined, but is particularly pertinent given that a malaria vaccine will be most beneficial in some of the world’s most resource-poor communities.

The choice of margin has important implications for the size of clinical trials; the narrower the margins, the larger the sample size required to have adequate statistical power or ability to reliably detect small clinically important differences between the first and second-generation malaria vaccines. A modest VE of a first-generation malaria vaccine will act favorably on superiority trial sample sizes of highly efficacious second-generation vaccines with large margins. However, in the case of less efficacious vaccines assessed in superiority, non-inferiority and equivalence trials narrow margins will have to be specified, which will have a profound impact on sample size. This is a major concern: will unfeasibly large sample sizes be a major obstacle in clinical testing of potential second-generation malaria vaccines?

### Are trials of second-generation malaria vaccines feasible?

**Figure 2:**
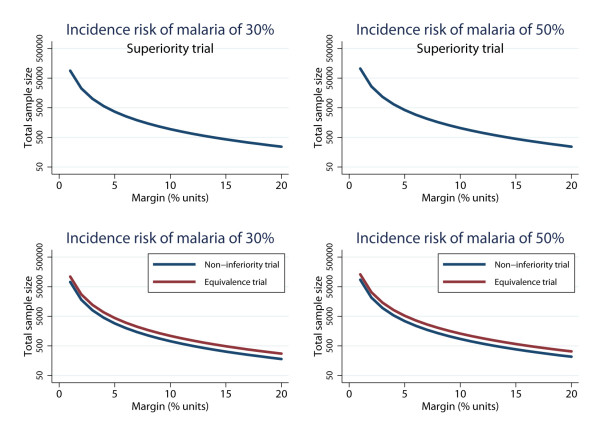
**Sample size estimates for superiority, non-inferiority and equivalence trials of second-generation malaria vaccines, according to different incidence risk of malaria in the first-generation vaccine group.** The figures show the estimated sample size required to detect a range of differences in the efficacy (margin %) between a second and first-generation malaria vaccine. The margin represents the absolute difference in the efficacy between the two vaccines for active-controlled trials; the relative difference for each value of absolute risk difference will therefore be greater for areas with a lower transmission (for example, absolute risk difference of 10% units equates to a relative risk of 0.67 and 0.80 when the baseline risk is 30% and 50%, respectively). The different incidence risks of malaria (proportion of individuals with malaria outcome during follow-up) in the first-generation vaccine groups corresponds to the approximate baseline risk observed in RTS,S phase II and III trials (that is, 30% and 50%) [4,5]. Sample sizes are calculated with 90% power at the 5% significance level by the authors using STATA (StataCorp; College Station, TX, USA). Note, as the incidence risk approaches 0.5, the standard error gets marginally larger; this explains why the sample size for the same absolute risk difference is bigger for a baseline risk of 50% compared with 30% (for example, total sample size required for a superiority trial with risk difference of 10% units is 778 and 1,030 for a baseline risk of 30% and 50%, respectively).

Sample size requirements of superiority, non-inferiority and equivalence trials will vary according to the assumed true difference between the second and first-generation malaria vaccine, the maximally tolerated difference required to reach a conclusion of superiority/non-inferiority/equivalence (Δ) and incidence of the malaria outcome [24]. Taking RTS,S as an example, and assuming a modest true VE of 30% or 50% [4,5], Figure 2 shows the range of sample sizes for differences in incidence risk of malaria between a new malaria vaccine and first-generation vaccine (for example, RTS,S) for superiority, non-inferiority and equivalence trials. The margin represents the absolute difference between the two vaccines; for non-inferiority and equivalence trials it is common practice to state a priori an 'absolute risk difference margin’ and base resulting sample size calculations on this absolute difference [25]. The figure shows that as the maximum acceptable clinical difference decreases the required sample size to determine superiority/non-inferiority/equivalence increases (Figure 2). The level of malaria transmission in a population has an impact on sample size, but counter intuitively, sample sizes will increase for a given absolute margin (Δ) when incidence risk of malaria (transmission) approaches 50% in active controlled trials (Figure 2). For example, a 5% margin and a baseline risk of 30% or 50%, gives sample size estimates for superiority trials of 3,678 and 4,182, for non-inferiority trials 2,878 and 3,426, and for equivalence trials 4,368 and 5,198, respectively.

The interpretation of the clinical significance of the absolute margin may vary according to the baseline risk of malaria (transmission), whereby the relative difference for each value of absolute risk difference will increase for lower transmission areas (for example, absolute risk difference of 10% units equates to a relative risk of 0.67 and 0.80 when the baseline risk is 30% and 50%, respectively). The baseline risk of malaria will vary across study sites influencing the sample size and the clinical interpretation of the difference between the two vaccines. Logically, trials will most likely be in higher transmission areas, where vaccines will have the biggest clinical impact, but differences in the underlying risk may also be influenced by the degree of pre-existing immunity, or the prevalence of the allele included in the new malaria vaccine in the parasite population. A pressing topical issue for consideration, even in the context of placebo-controlled superiority trials, is declining malaria transmission, particularly in sites that have the capacity to perform randomized controlled trials [26]. This will impact on the feasibility of conducting trials and the type of trials performed. Failing to take into consideration other factors that contribute to malaria risk has significant implications such that trials may be underpowered and incorrect conclusions about the VE of the second-generation malaria vaccine may be reached.

There is an expectation that second-generation malaria vaccines with substantially greater efficacy than first-generation vaccines will ultimately be developed. These more efficacious vaccines would be tested in a superiority trial versus the first-generation vaccine. Using parameters from the RTS,S clinical trials, if we assume a VE of up to 50% for the first-generation vaccine and a VE of 80% for the new vaccine [3] (that is, a 30% margin) then a sample size of just 96 would be required to demonstrate superiority (Figure 2B). If the superiority margin for a new vaccine was set at least 10% higher than the licensed vaccine, which is probably the minimum level that would be considered acceptable by the community, then the required sample size would be 1,030. These estimates suggest that testing more efficacious new vaccines in superiority trials, including placebo-controlled trials of new malaria vaccines, would be highly feasible since many clinical trials of this size have been performed in malaria-endemic countries.

For non-inferiority and equivalence trials the situation is very different. For non-inferiority trials that are powered to detect a difference of 3%, 5% or 10% in malaria risk between the new vaccine and existing vaccine (for example, RTS,S), 9,516, 3,462 and 1,058 participants, respectively, would be needed (Figure 2B). For equivalence trials, sample size estimates are higher with a requirement for 14,440, 5,198 and 1,300 participants, respectively (Figure 2D). These estimates indicate that performing non-inferiority and equivalence trials would require a much greater investment of resources, and may require multiple study sites to recruit sufficient participants into the trials. This would be the case for vaccines that seek to add to the first-generation vaccine without increasing its efficacy, such as a combination vaccine including activity against an additional lifecycle stage or activity against *P. vivax*. The current phase III trial of RTS,S has 15,460 participants and large prophylactic antimalarial equivalence trials, with extended follow-up to determine incidence risk of malaria, in 1,600 participants have been performed [23], indicating that large clinical trials of malaria vaccines are possible in malaria-endemic regions. However, there will be a limit to how many of these trials can be performed. It may also be difficult to find a sufficiently large population of individuals willing to forgo receiving a vaccine believed to be beneficial for the duration of the trial. Significant community engagement and appropriate scientific communication will be instrumental for the development, evaluation, and possible deployment of a second-generation malaria vaccine.

While hurdles in sample size may be overcome, the malaria community must also come together to define methodology to ensure the feasibility of second-generation vaccine trials, particularly vaccines that target different lifecycle stages. For pre-erythrocytic vaccines, such as RTS,S, there is a broad consensus on clinical definitions and endpoints and the analytical methodology for measuring the reduction in morbidity [27], but there is no such consensus for blood-stage vaccines or trial designs to determine the reduction of infectivity of humans for mosquitoes (for transmission blocking vaccines) [8] or trial methodology for a *P. vivax* vaccine. It is essential that comparison trials consider the differences in clinical endpoints as well as the duration and mode of protection because this may also influence decisions on whether vaccines are tested in combination or as standalone vaccines, which will impact on the aforementioned design and size of the vaccine trial. Calculations of RTS,S VE have waned with time [28] and been shown to differ if different assumptions of the mode of protection are made. RTS,S is assumed to be a 'leaky’ vaccine (which offers partial protection by reducing the risk of infection from each exposure) [29] as demonstrated by convergence of survival curves of placebo and RTS,S groups. The preliminary phase III trial data showed that VE of RTS,S was 56% when calculated using time-to-event methodology (1 minus the hazard rate ratio) suitable for this mode of protection, but RTS,S VE was reduced to 36% when assumed to provide 'all or nothing’ protection (that is, calculated as 1 minus the risk ratio) [30]. While additional factors may contribute to differences in VE estimates, such as transmission heterogeneity and waning efficacy over time, it is clear that a consensus must be reached for defining clinical endpoints and methodology so that the VE of different malaria vaccines can be compared both within and across trials.

### Implementing a second-generation vaccine into public health practice

Once methodological issues are overcome, and a second-generation vaccine is shown to be favorable to the first-generation vaccine, the results may still fail to clarify to policy makers whether new malaria vaccines are sufficiently effective to justify inclusion in public health vaccination campaigns [31]. For example several vaccines towards *Haemophilus influenza* type b (Hib), cholera and typhoid fever were shown to be safer and at least protective as the traditional active control vaccine, but were met with uncertainty or ambivalence in discussions about their implementation in public health programs in low-income and middle-income countries [31]. The reasons for which are many, but it is imperative that protective malaria vaccines, whether second or first generation, are not hindered by this process and are made available to those who need it at the earliest opportunity.

## Summary

This opinion piece highlights important issues that need to be considered in developing and testing second-generation malaria vaccines in the future. Selecting which new malaria vaccines go forward, and defining appropriate methodology for assessment in clinical trials, is crucial. Analyses suggest that clinical trials of new malaria vaccines that have greater efficacy than partially effective malaria vaccines require modest sample sizes and are highly feasible. However, testing vaccines of similar efficacy in non-inferiority or equivalence trials would be logistically more challenging and require substantial human and financial resources. Furthermore, it is important that we identify immunological correlates of malaria to enable the assessment of new malaria vaccines through immunological assays, thereby aiding vaccine development, and determine methodology for examining new malaria vaccines containing different lifecycle stages or *Plasmodium* spp. Poorly designed trials of second-generation vaccines may mean that clinically beneficial vaccines are rejected or inferior malaria vaccines are adopted. It is critical that the malaria community keep moving forward and overcome these obstacles to ensure the feasibility of the development, and licensure, of second-generation malaria vaccines. This will ensure that the most practical efficacious vaccine can be implemented into public health practice to reduce, and potentially eliminate, the burden of disease and death caused by malaria.

## Abbreviations

VE: Vaccine efficacy.

## Competing interests

The authors declare they have no conflicts of interest.

## Authors’ contributions

All authors wrote, read and approved the final manuscript.

## Pre-publication history

The pre-publication history for this paper can be accessed here:

http://www.biomedcentral.com/1741-7015/11/232/prepub

## References

[B1] Murray CJ Rosenfeld LC Lim SS Andrews KG Foreman KJ Haring D Fullman N Naghavi M Lozano R Lopez AD Global malaria mortality between 1980 and 2010: a systematic analysis Lancet 2012 379 413 431 22305225 10.1016/S0140-6736(12)60034-8

[B2] WHO World Malaria Report 2012 2012 Geneva, Switzerland: WHO Press/World Health Organization 1 89

[B3] Malatia Vaccine Initiative Malaria Vaccine Technology Roadmap http://www.malariavaccine.org/files/Malaria_Vaccine_TRM_Final_000.pdf

[B4] Agnandji ST Lell B Soulanoudjingar SS Fernandes JF Abossolo BP Conzelmann C Methogo BG Doucka Y Flamen A Mordmüller B Issifou S Kremsner PG Sacarlal J Aide P Lanaspa M Aponte JJ Nhamuave A Quelhas D Bassat Q Mandjate S Macete E Alonso P Abdulla S Salim N Juma O Shomari M Shubis K Machera F Hamad AS Minja R First results of phase 3 trial of RTS, S/AS01 malaria vaccine in African children New Engl J Med 2011 365 1863 1875 22007715 10.1056/NEJMoa1102287

[B5] Agnandji ST Lell B Fernandes JF Abossolo BP Methogo BG Kabwende AL Adegnika AA Mordmüller B Issifou S Kremsner PG Sacarlal J Aide P Lanaspa M Aponte JJ Machevo S Acacio S Bulo H Sigauque B Macete E Alonso P Abdulla S Salim N Minja R Mpina M Ahmed S Ali AM Mtoro AT Hamad AS Mutani P RTS,S Clinical Trials Partnership A phase 3 trial of RTS,S/AS01 malaria vaccine in African infants N Engl J Med 2012 2012 2012 10.1056/NEJMoa1208394 PMC10915853 23136909

[B6] Thera MA Doumbo OK Coulibaly D Laurens MB Ouattara A Kone AK Guindo AB Traore K Traore I Kouriba B Diallo DA Diarra I Daou M Dolo A Tolo Y Sissoko MS Niangaly A Sissoko M Takala-Harrison S Lyke KE Wu Y Blackwelder WC Godeaux O Vekemans J Dubois MC Ballou WR Cohen J Thompson D Dube T Soisson L A field trial to assess a blood-stage malaria vaccine New Engl J Med 2011 365 1004 1013 21916638 10.1056/NEJMoa1008115 PMC3242358

[B7] Genton B Betuela I Felger I Al-Yaman F Anders RF Saul A Rare L Baisor M Lorry K Brown GV Pye D Irving DO Smith TA Beck HP Alpers MP A recombinant blood-stage malaria vaccine reduces *Plasmodium falciparum* density and exerts selective pressure on parasite populations in a phase 1-2b trial in Papua New Guinea J Infect Dis 2002 185 820 827 11920300 10.1086/339342

[B8] Schwartz L Brown GV Genton B Moorthy VS A review of malaria vaccine clinical projects based on the WHO rainbow table Malar J 2012 11 11 22230255 10.1186/1475-2875-11-11 PMC3286401

[B9] McCarthy JS Marjason J Elliott S Fahey P Bang G Malkin E Tierney E Aked-Hurditch H Adda C Cross N Richards JS Fowkes FJ Boyle MJ Long C Druilhe P Beeson JG Anders RF A phase 1 trial of MSP2-C1, a blood-stage malaria vaccine containing 2 isoforms of MSP2 formulated with Montanide(R) ISA 720 PLoS ONE 2011 6 e24413 21949716 10.1371/journal.pone.0024413 PMC3176224

[B10] Greenwood BM Targett GA Malaria vaccines and the new malaria agenda Clin Microbiol Infect 2011 17 1600 1607 21883665 10.1111/j.1469-0691.2011.03612.x

[B11] Greenwood B Immunological correlates of protection for the RTS, S candidate malaria vaccine Lancet Infect Dis 2011 11 75 76 21237716 10.1016/S1473-3099(11)70001-9

[B12] Kester KE Cummings JF Ofori-Anyinam O Ockenhouse CF Krzych U Moris P Schwenk R Nielsen RA Debebe Z Pinelis E Juompan L Williams J Dowler M Stewart VA Wirtz RA Dubois MC Lievens M Cohen J Ballou WR Heppner DG Jr RTS, S Vaccine Evaluation Group Randomized, double-blind, phase 2a trial of falciparum malaria vaccines RTS, S/AS01B and RTS, S/AS02A in malaria-naive adults: safety, efficacy, and immunologic associates of protection J Infect Dis 2009 200 337 346 19569965 10.1086/600120

[B13] Casares S Brumeanu TD Richie TL The RTS, S malaria vaccine Vaccine 2010 28 4880 4894 20553771 10.1016/j.vaccine.2010.05.033

[B14] Ndungu FM Mwacharo J Kimani D Kai O Moris P Jongert E Vekemans J Olotu A Bejon P A statistical interaction between circumsporozoite protein-specific T cell and antibody responses and risk of clinical malaria episodes following vaccination with RTS, S/AS01E PLoS ONE 2012 7 e52870 23300801 10.1371/journal.pone.0052870 PMC3531328

[B15] Postema AS Myers MG Breiman RF Challenges in the development, licensure, and use of combination vaccines Clin Infect Dis 2001 33 S261 S266 11709757 10.1086/322560

[B16] Scott IA Non-inferiority trials: determining whether alternative treatments are good enough Med J Aust 2009 190 326 330 19296815 10.5694/j.1326-5377.2009.tb02425.x

[B17] The Four Artemisinin-Based Combinations (4ABC) Study Group A head-to-head comparison of four artemisinin-based combinations for treating uncomplicated malaria in African children: a randomized trial PLoS Med 2011 8 e1001119 22087077 10.1371/journal.pmed.1001119 PMC3210754

[B18] Tshefu AK Gaye O Kayentao K Thompson R Bhatt KM Sesay SS Bustos DG Tjitra E Bedu-Addo G Borghini-Fuhrer I Duparc S Shin CS Fleckenstein L Pyronaridine-artesunate Study Team Efficacy and safety of a fixed-dose oral combination of pyronaridine-artesunate compared with artemether-lumefantrine in children and adults with uncomplicated Plasmodium falciparum malaria: a randomised non-inferiority trial Lancet 2010 375 1457 1467 20417857 10.1016/S0140-6736(10)60322-4

[B19] Bassat Q Mulenga M Tinto H Piola P Borrmann S Menéndez C Nambozi M Valéa I Nabasumba C Sasi P Bacchieri A Corsi M Ubben D Talisuna A D’Alessandro U Dihydroartemisinin-piperaquine and artemether-lumefantrine for treating uncomplicated malaria in African children: a randomised, non-inferiority trial PLoS ONE 2009 4 e7871 19936217 10.1371/journal.pone.0007871 PMC2776302

[B20] Premji Z Umeh RE Owusu-Agyei S Esamai F Ezedinachi EU Oguche S Borrmann S Sowunmi A Duparc S Kirby PL Pamba A Kellam L Guiguemdé R Greenwood B Ward SA Winstanley PA Chlorproguanil-dapsone-artesunate versus artemether-lumefantrine: a randomized, double-blind phase III trial in African children and adolescents with uncomplicated Plasmodium falciparum malaria PLoS ONE 2009 4 e6682 19690618 10.1371/journal.pone.0006682 PMC2724683

[B21] Sagara I Rulisa S Mbacham W Adam I Sissoko K Maiga H Traore OB Dara N Dicko YT Dicko A Djimdé A Jansen FH Doumbo OK Efficacy and safety of a fixed dose artesunate-sulphamethoxypyrazine-pyrimethamine compared to artemether-lumefantrine for the treatment of uncomplicated falciparum malaria across Africa: a randomized multi-centre trial Malar J 2009 8 63 19366448 10.1186/1475-2875-8-63 PMC2678145

[B22] Sirima SB Tiono AB Gansane A Diarra A Ouedraogo A Konate AT Kiechel JR Morgan CC Olliaro PL Taylor WR The efficacy and safety of a new fixed-dose combination of amodiaquine and artesunate in young African children with acute uncomplicated *Plasmodium falciparum* Malar J 2009 8 48 19291301 10.1186/1475-2875-8-48 PMC2662869

[B23] Briand V Bottero J Noël H Masse V Cordel H Guerra J Kossou H Fayomi B Ayemonna P Fievet N Massougbodji A Cot M Intermittent treatment for the prevention of malaria during pregnancy in Benin: a randomized, open-label equivalence trial comparing sulfadoxine-pyrimethamine with mefloquine J Infect Dis 2009 200 991 1001 19656069 10.1086/605474

[B24] Rao MR Blackwelder WC Troendle JF Naficy AB Clemens JD Sample size determination for phase II studies of new vaccines Vaccine 2002 20 3364 3369 12213406 10.1016/s0264-410x(02)00317-1

[B25] Piaggio G Elbourne DR Altman DG Pocock SJ Evans SJ Reporting of noninferiority and equivalence randomized trials: an extension of the CONSORT statement JAMA 2006 295 1152 1160 16522836 10.1001/jama.295.10.1152

[B26] O’Meara WP Mwangi TW Williams TN McKenzie FE Snow RW Marsh K Relationship between exposure, clinical malaria, and age in an area of changing transmission intensity Am J Trop Med Hygiene 2008 79 185 191 PMC2547116 18689622

[B27] Moorthy V Reed Z Smith PG Measurement of malaria vaccine efficacy in phase III trials: report of a WHO consultation Vaccine 2007 25 5115 5123 17577487 10.1016/j.vaccine.2007.01.085

[B28] Olotu A Fegan G Wambua J Nyangweso G Awuondo KO Leach A Lievens M Leboulleux D Njuguna P Peshu N Marsh K Bejon P Four-year efficacy of RTS, S/AS01E and its interaction with malaria exposure N Engl J Med 2013 368 1111 1120 23514288 10.1056/NEJMoa1207564 PMC5156295

[B29] Lievens M Aponte JJ Williamson J Mmbando B Mohamed A Bejon P Leach A Statistical methodology for the evaluation of vaccine efficacy in a phase III multi-centre trial of the RTS, S/AS01 malaria vaccine in African children Malar J 2011 10 222 21816030 10.1186/1475-2875-10-222 PMC3167766

[B30] Duncan CJ Hill AV What is the efficacy of the RTS, S malaria vaccine? BMJ Clin Res Ed 2011 343 d7728 10.1136/bmj.d7728 PMC3287289 22167776

[B31] Clemens J Brenner R Rao M Tafari N Lowe C Evaluating new vaccines for developing countries. Efficacy or effectiveness? JAMA 1996 275 390 397 8569019

